# Characterization of three novel *Helicobacter* species infecting stomachs of dogs and cats: *Helicobacter gastrocanis* sp. nov., *Helicobacter gastrofelis* sp. nov., and *Helicobacter felistomachi* sp. nov.

**DOI:** 10.3389/fmicb.2024.1459401

**Published:** 2025-01-17

**Authors:** Emiko Rimbara, Sae Aoki, Masato Suzuki, Hirotaka Kobayashi, Taisuke Nakagawa, Yuko Goto-Koshino, Sachiyo Nomura, Wan-Ying Du, Hidenori Matsui, Shigetarou Mori, Keigo Shibayama, Tsuyoshi Kenri, Koichi Ohno

**Affiliations:** ^1^Department of Bacteriology II, National Institute of Infectious Diseases, Tokyo, Japan; ^2^Antimicrobial Resistance Research Center, National Institute of Infectious Diseases, Tokyo, Japan; ^3^Department of Pathology, National Institute of Infectious Diseases, Tokyo, Japan; ^4^Veterinary Medical Center, Graduate School of Agricultural and Life Sciences, The University of Tokyo, Tokyo, Japan; ^5^Department of Gastrointestinal Surgery, Graduate School of Medicine, The University of Tokyo, Japan; ^6^Department of Clinical Pharmaceutical Sciences, Hoshi University, Tokyo, Japan; ^7^Department of Bacteriology, Nagoya University Graduate School of Medicine, Nagoya, Japan

**Keywords:** gastric *Helicobacter* species, cats, dogs, novel species, gastric disease

## Abstract

*Helicobacter* species infecting the stomachs of dogs and cats are potentially pathogenic and have been isolated from patients with gastric diseases. In the present study conducted in Japan, among the nine *Helicobacter* strains that we isolated from dogs and cats, NHP19-003^T^ from a dog, and strains NHP19-012^T^ and NHP21-005^T^ from cats were identified to be the strains most closely related to *Helicobacter heilmannii* ASB1^T^ based on a 16S rRNA comparison (98.7–99.2% similarity with *H. heilmannii* ASB1^T^). However, none of their whole genomes showed more than average nucleotide identity (ANI) threshold value (95–96%) to any *Helicobacter* species (85.1, 86.7, and 86.6% ANI, respectively, with *H. heilmannii* ASB1^T^), including when compared to each other. Furthermore, NHP19-003^T^, NHP19-012^T^, and NHP21-005^T^ exhibited protein profiles different from known gastric *Helicobacter* species, as revealed by MALDI-TOF MS, indicating that they are novel *Helicobacter* species. We, thus, propose these novel *Helicobacter* species as follows: *Helicobacter gastrocanis* sp. nov. (type strain NHP19-003^T^ [=JCM 39159^T^ = DSM 111619^T^]), *Helicobacter gastrofelis* sp. nov. (type strain NHP19-012^T^ [=JCM 39160^T^]) and *Helicobacter felistomachi* sp. nov. (type strain NHP21-005^T^ [=JCM 39513^T^]). These novel strains have respective GC content values of 48.3, 46.9, and 47.1%. Phylogenetic analysis based on *ureAB* gene sequences obtained from gastric specimens from 47 dogs and 24 cats in Japan revealed that 29.8% of dogs were infected with *H. gastrocanis*, while *H. gastrofelis* infected 44.7% of dogs and 12.5% of cats. Additionally, 10.6% of dogs and 20.8% of cats were infected with *H. felistomachi*. Animal experiments have confirmed that these three novel species elicit gastric inflammatory responses. This study findings reveal the prevalence of novel gastric *Helicobacter* species in dogs and cats in Japan and their pathogenicity.

## Introduction

*Helicobacter pylori* was discovered as a pathogen of the human stomach by Robin Warren and Barry Marshall in 1983, followed by the establishment of the genus *Helicobacter* in 1989 (Goodwin et al., 1989). *Helicobacter* species infecting the stomach are called gastric *Helicobacter* that neutralizes around itself by producing urease in order to survive in acidic condition. Gastric *Helicobacter* species infects various animals, including dolphins, whales, pigs, cats, dogs, non-human primates, and humans. All these exhibits a corkscrew-like spiral form different from that of *H. pylori. H. pylori* is known to infect the human stomach and cause gastric diseases such as gastric cancer, while non-*H. pylori* gastric *Helicobacter* species such as *Helicobacter suis* infecting pigs and *Helicobacter ailurogastricus* infecting cats have been reported as possible causes of gastric diseases such as gastric mucosa associated lymphoid tissue (MALT) lymphoma and gastric ulcers in human (Rimbara et al., 2021; Sano et al., 2023).

*Helicobacter* species include 54 species so far, and among them, the following seven gastric *Helicobacter* species have been identified in dogs and cats: *H. ailurogastricus* (Joosten et al., 2016), *Helicobacter baculiformis* (Baele et al., 2008), *Helicobacter bizzozeronii* (Hänninen et al., 1996), *Helicobacter cynogastricus* (Van den Bulck et al., 2006), *Helicobacter felis* (Paster et al., 1991), *Helicobacter heilmannii* (Smet et al., 2012), and *Helicobacter salomonis* (Jalava et al., 1997). Previous study on gastric *Helicobacter* infecting dogs and cats in Japan suggested that *Helicobacter* species infect Japanese dogs and cats include the species, which are not identifiable with known gastric *Helicobacter* species. Although culture is necessary for species identification, cultured strain was not obtained in the previous study. We have recently established methods to isolate *H. suis* and *H. ailurogastricus* from human stomachs (Rimbara et al., 2021; Sano et al., 2023). In this study, we isolated and cultured gastric *Helicobacter* species infecting dogs and cats in Japan, and analyzed the genome of the isolates. As a result, we isolated nine gastric *Helicobacter* species strains, including three novel species, from dogs and cats suffering from gastrointestinal disease in 2019 and 2021 in Japan. According to the recent standards for the identification of novel *Helicobacter* species proposed by On et al. (2017) and Riesco and Trujillo (2024) we verify the phenotypic, phylogenetic and genotypic characterization of these strains. Based on these results, we propose the names *Helicobacter gastrocanis* sp. nov., *Helicobacter gastrofelis* sp. nov. and *Helicobacter felistomachi* sp. nov. for the three novel species.

## Materials and methods

### Strains and morphology studies

Nine strains isolated from pets that were visited the Veterinary Medical Center, The University of Tokyo between 2019 and 2022 were analyzed in this study. Pets suffering from various conditions such as hypoalbuminemia and chronic vomiting underwent endoscopic examination and endoscopically obtained gastric specimens were used for the isolation of *Helicobacter* species when the characteristic corkscrew-like bacterium was observed by Giemsa staining. The isolation of *Helicobacter* species was performed according to a previously reported method (Rimbara et al., 2021). In brief, these specimens were homogenized in Brucella Broth with 0.05% HCl (pH around 5) and spread on non-*Helicobacter pylori Helicobacter* (NHPH) agar plates containing 1.5% agar (Difco Becton Dickinson) and NHPH medium consisting of Brucella broth (Difco Becton Dickinson), 20% fetal bovine serum (Gibco), Vitox supplement (Oxoid), Campylobacter-selective supplement, Skirrow (Oxoid), 5 mg/mL amphotericin B (FUJIFILM Wako), and 0.05% HCl (FUJIFILM Wako). The plates were incubated for 6 to 14 days at 37°C under microaerobic conditions with a gas mixture of 5% O_2_ and 12% CO_2_. A single colony was sub-cultured on the NHPH agar plates, followed by inoculation into the two-layer NHPH medium. The biopsy specimen taken for clinical diagnostic purpose was used for this study. For characterization of the strain according to its morphological features, we performed scanning electron micrograph and Giemsa staining (Rimbara et al., 2021).

### Genome sequencing

Whole-genome sequencing of all strains was performed using the MiniSeq (NHP19-002, NHP19-003^T^, NHP19-009, NHP19-012^T^, NHP21-005^T^, NHP21-011), HiSeq (NHP20-010, NHP20-013), and NovaSeq (NHP22-001) platforms (Illumina, San Diego, USA). Genomic DNA from strains was extracted using DNeasy Blood & Tissue Kit (Qiagen) according to the manufacturer’s instructions. The library for Illumina sequencing (paired-end, insert size of 500–900 bp) was prepared using the Nextera XT DNA Library Prep Kit (Illumina, San Diego, USA). Illumina reads were assembled using Shovill v1.1.0. and default parameters, to acquire the draft genome sequences. For NHP19-003^T^, NHP19-012^T^, and NHP21-005^T^, which were considered novel species, complete genomes were determined on the MinION platform (Oxford Nanopore Technologies [ONT], Oxford, UK). Genomic DNA from isolated strains was extracted using Genomic-tips 20/G and buffers (Qiagen) according to the manufacturer’s instructions. The library for MinION sequencing was prepared using Rapid Sequencing Kit (SQK-RBK004) (ONT, Oxford, UK). ONT reads were base-called using Guppy v4.2.2 (NHP19-003^T^ and NHP19-012^T^) or v6.4.2 (NHP21-005^T^) and were assembled *de novo* using Canu v1.8 (NHP19-003^T^ and NHP19-012^T^) or v2.1 (NHP21-005^T^)^1^ (Koren et al., 2017). The overlap region in the assembled contig was detected using LAST^2^ (Frith et al., 2010) and was trimmed manually. Illumina reads were mapped onto the resulting circular sequences, and sequencing errors were corrected twice with ONT reads using Racon v1.4.13^3^ (Vaser et al., 2017), and then polished twice with Illumina reads combined using Pilon v1.20.1^4^ (Walker et al., 2014), resulting in the complete genomes. The obtained complete and draft genomic sequences were annotated using the DFAST server^5^.

The average nucleotide identity (ANI) values were calculated using Pyani 0.2.12^6^ (Pritchard et al., 2016). Since it has been reported that a value of 70% DNA–DNA hybridization for species delineation corresponds to about 95% ANI, 95% ANI cut-off values were used for species identification (Goris et al., 2007). According to the standard recommended by Riesco and Trujillo (2024), the average amino acid identity (AAI) values were calculated using EzAAI^7^ (Kim et al., 2021). Digital DNA–DNA hybridization (dDDH) values were calculated using GGDC^8^ and 70% dDDH cut-off values were used for species identification (Meier-Kolthoff et al., 2013).

### Phylogenetic characteristics

The alignment of the VacA-like proteins was generated by MAFFT version 7.49 and the tree was constructed using RAxML-NG version 1.1.0 with a LG + G4 model and 1,000 bootstrap replicates. The accession numbers of the VacA-like proteins of the *Helicobacter* species used in the analysis are summarized in Supplementary Table S1.

A phylogenetic analysis using a core-gene and 16S rRNA sequence alignment, was generated using Roary (Page et al., 2015) and MAFFT version 7.49 (Katoh et al., 2002), respectively. Aligned sequences were used for phylogenetic reconstruction using RAxML-NG version 1.1.0 with a GTR + G + I model and 1,000 bootstrap replicates (Kubota-Aizawa et al., 2017a; Kubota-Aizawa et al., 2017b). The genomic information and accession numbers of the 16S rRNA and genomic sequences of the known *Helicobacter* species used in the analysis are summarized in Supplementary Tables S2, S3.

The *ureAB* sequences of *Helicobacter* species amplified and sequenced from gastric biopsies of dogs (47 samples) and cats (24 samples) in Japan, which were obtained in previous studies (Kubota-Aizawa et al., 2017a; Kubota-Aizawa et al., 2017b), were re-analyzed in this study. Sequences obtained in previous studies were assigned “LCXXXXXXXX” as accession numbers. The accession numbers of the *ureAB* sequences used in the analysis are summarized in Supplementary Table S4. The alignment of the partial *ureA* and *ureB* genes was generated by MAFFT version 7.49 and the tree was constructed using RAxML-NG version 1.1.0 with a GTR + G + I model and 1,000 bootstrap replicates.

### Phenotypic characteristics

The physiological characteristics of NHP19-003^T^, NHP19-012^T^ and NHP21-005^T^ were demonstrated using API Campy (bioMérieux) according to the manufacturer’s instruction. Tolerance to 1% bile, 1% glycine, and 1.5% NaCl was assessed by culturing organisms on NHPH agar plates supplemented with each compound. The plates were inoculated with 10 μL cultures prepared according to McFarland standard 6.0 (bioMérieux) and incubated for 7 days at 37°C under microaerobic conditions. Bacterial growth was visually confirmed. Catalase activity of the isolates was examined by adding a 3% H_2_O_2_ solution and observing the reaction within 5 s. Oxidase activity was determined using the Poremedia® oxidase test indicator (Eiken Chemical). To clarify the optimum temperature, strains were incubated at 25°C, 37°C, and 42°C under microaerobic conditions. Oxygen requirements were tested at 37°C under aerobic, microaerobic, and anaerobic conditions.

### MALDI-TOF MS assays

Protein profiles were generated from MALDI Biotyper (BrukerBiotyper, BrukerDaltonics). Bacterial isolates were prepared according to an acetonitrile–formic acid-extraction protocol provided by the manufacturer and analyzed using flexAnalysis software (version 3.4; BrukerDaltonics). MALDI-TOF mass spectra were recorded with an LT microflex mass spectrometer (BrukerDaltonics) within the range of 2,000 Da to 20,000 Da according to the manufacture, m/z stands for mass to charge ratio.

### Animal experiments

Mice infections were performed using three-week-old female C57BL/6 N mice (CLEA Japan, Inc. Tokyo, Japan). Three novel *Helicobacter* sp. strains (NHP19-003^T^, NHP19-012^T^, and NHP21-005^T^), *H. ailurogastricus* ASB7^T^, and *H. suis* NHP19-4004 were infected intragastrically using a feeding needle with 0.2 mL of 1 × 10^9^ colony-forming units (CFU) ml^−1^ of every other day, repeated three times. Five mice were infected for each strain. After 1 month, mice were sacrificed, and the stomachs were cut along the greater curvature and the gastric content were removed and rinsed with PBS. Half of the stomach was used for histopathological examination and the other half for DNA extraction. For histopathology the stomach was fixed with 10% (wt/vol) neutral-buffered formalin (FUJIFILM Wako), embedded in paraffin, and sectioned to approximately 4 μm thickness. The sections were stained with hematoxylin–eosin (H&E) and immunohistochemically stained for CD3(+) and CD19(+) using specific antibodies. Paraffin sections (4 μm) were deparaffined, rehydrated, antigen-retrieved in the Immunosaver (Nishin EM, Tokyo, Japan), antigen-retrieved, blocked, and incubated with the primary antibodies (anti-CD3, Cell Signaling #99940, 1/600dilution; CD19, Cell Signaling #90176, 1/3200 dilution) overnight at 4°C. The antigen–antibody complexes on the slides were visualized using VECTASTAIN Elite ABC HRP Kit and DAB Substrate Solution (#SK-4105, VECTOR). Finally, cell nuclei were stained with hematoxylin (MUTO Pure Chemicals Co., Ltd., Japan). For the fundic and pyloric regions, 30 glands were randomly selected for each sample, and quantification was performed by counting the number of positive cells within these glands to determine the ratio. For the forestomach-glandular border, the number of positive cells in the border was counted and the result was categorized as follows: <20 positive cells was 1, 20–100 positive cells was 2, and >100 positive cells was 3.

The relative *Helicobacter* sp. count in the mouse stomachs was evaluated by the comparative ΔCt method using probe-based quantitative PCR targeting the region of 16S rRNA specific to NHPH species as shown in a previous report (Matsui et al., 2023). *β*-actin was used as an internal control using the following primers: β-actin_forward (5’-TGAAGTGTGACGTTGACATCC-3′), β-actin_reverse (5′-TCCTTCTGCATCCTGTCAGC-3′), and β-actin_probe (/56-FAM/ATTCCATAC/ZEN/CCAAGAAGGAAGGCTGG/3IABkFQ/). DNA was extracted from the mouse stomach using the DNeasy Blood & Tissue Kit (Qiagen) and PCRs were performed in an Applied Biosystems 7500 Fast Real-Time PCR System (Thermo Fisher Scientific). Probe-PCR was performed in duplicate for each sample, and the 2^-ΔCt^ (16S-β-actin) value of each sample was used for comparison. The relative value was calculated by dividing the 2^-ΔCt^ (16S-β-actin) of each sample by the average of the 2^-ΔCt^ (16S-β-actin) values of the *H. ailurogastricus* ASB7-infected group. All animal experiments and the study protocol were approved by the Committee for Animal Experimentation of the National Institute of Infectious Diseases (approval number: 123036).

### Statistical analysis

For data on the number of CD3(+) and CD19(+) cells that violated parametric assumptions such as normality and homogeneity of variances, the Kruskal–Wallis test was employed, with *post hoc* analysis conducted using Dunn’s multiple comparison test. Means were adjusted by ± one standard deviation (SD). In an RNA assay, each *Helicobacter* sp.-infected group was compared with the ASB7^T^-infected group using one-way ANOVA with Tukey’s test. Prism 10 (GraphPad Software, La Jolla, CA, USA) was used for statistical analysis. Data represent means ± SD. *p* < 0.05 was considered statistically significant.

## Results

### *Helicobacter* species identified by genomic analysis

**Figure 1:**
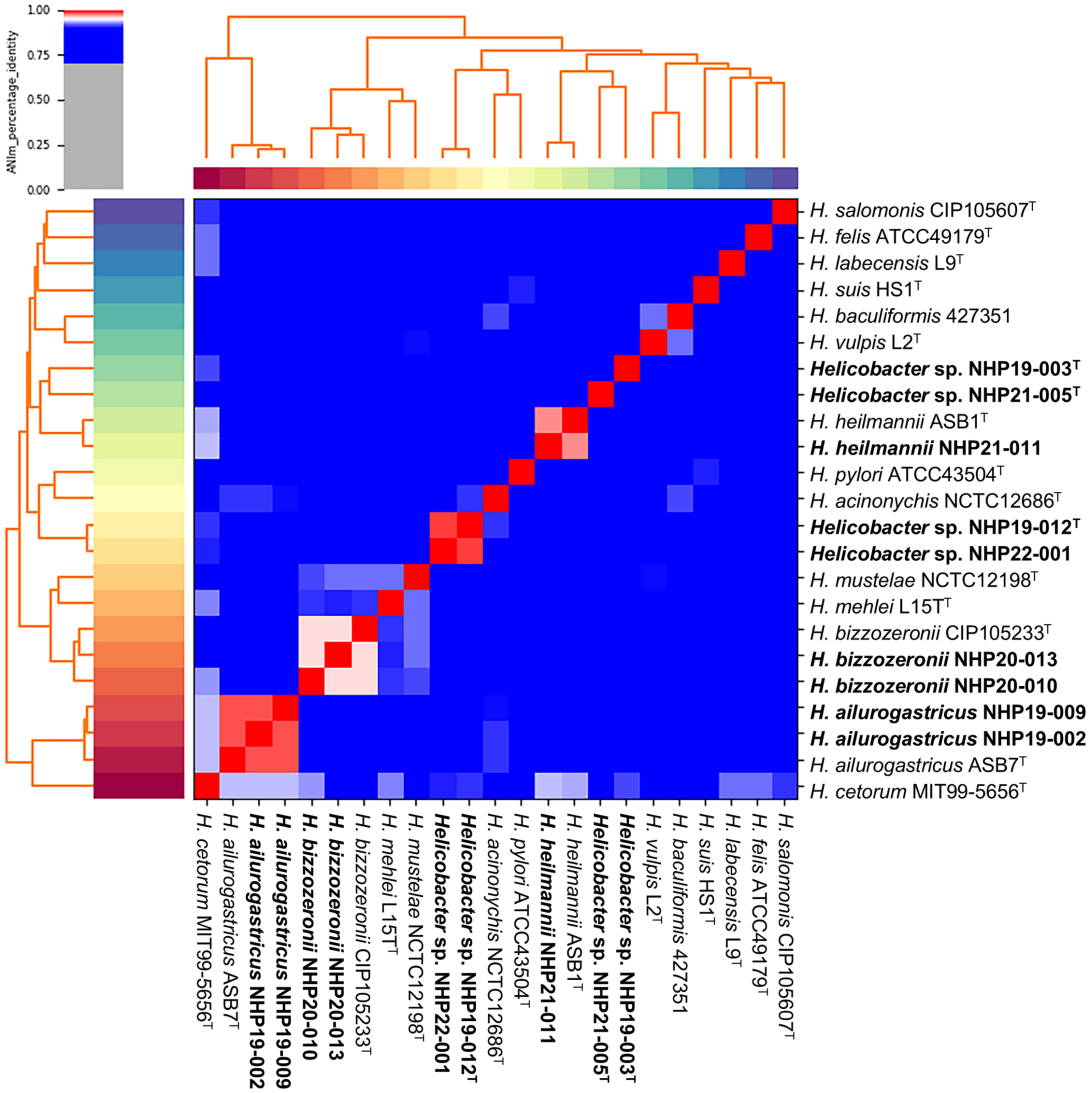
Average nucleotide identity (ANI) of the nine strains analyzed in this study compared to other *Helicobacter* species. A heatmap and hierarchical clustering are shown. Pairs of genomes with ANI > 95% can be considered as from the same species. The nine strains shown in bold are those isolated in this study.

The list of the nine strains isolated in this study are shown in Table 1. The nine strains of *Helicobacter* species obtained were isolated from different individual animals. Among the nine strains, two strains [NHP19-002 (Accession No. GCA_030270085.1) and NHP19-009 (Accession No. GCF_030270105.1)] were identified as *Helicobacter ailurogastricus*, two strains [NHP20-010 (Accession No. GCF_030270125.1) and NHP20-013(Accession No. GCA_036248205.1)] were *Helicobacter bizzozeronii*, and NHP21-011 (Accession No. GCF_030270145.1) was *Helicobacter heilmannii* by the average nucleotide identity (ANI) value calculation using whole genomic sequences. The four remaining strains (NHP19-003^T^, NHP19–012^T^, NHP21-005^T^, and NHP22-001) had no more than ANI threshold value (95–96%) with any *Helicobacter* species, suggesting that these strains are novel *Helicobacter* species (Figure 1 and Table 1). The ANI value between NHP19-012^T^ and NHP22-001 was 98.5%, suggesting that these two strains were of the same species (Table 1). Therefore, subsequent experiments were conducted using only the three representative strains: NHP19-003^T^, NHP19-012^T^, and NHP 21-005^T^. It was also clear that NHP19-003^T^, NHP19-012^T^, and NHP21-005^T^ are distinct from any *Helicobacter* species, since the ANI value between these strains were 85.7% (NHP19-003^T^ and NHP19-012^T^), 86.5% (NHP19-003^T^ and NHP21-005^T^), and 86.5% (NHP19-012^T^ and NHP21-005^T^), respectively. The average amino acid identity (AAI) values among NHP19-003^T^, NHP19-012^T^, NHP21-005^T^, *H. heilmannii* ASB1^T^, and *H. ailurogastricus* ASB7^T^ ranged between 87.0 and 89.8%. These values are lower than the AAI values of 90.2% between *H. pylori* ATCC43504^T^ and *H. acynonychis* NCTC12686^T^, and 92.6% between *H. bizzozeronii* CCUG35545^T^ and *H. mehei* L15^T^. This supports the conclusion that NHP19-003^T^, NHP19-012^T^, and NHP21-005^T^ are three novel *Helicobacter* species (Table 1). The digital DNA–DNA Hybridization (dDDH) analysis also indicated that these three strains are novel *Helicobacter* species as well as ANI and AAI value (Table 1).

**Table 1:** Strains isolated in this study.

Strain	Year	Origin	Diseases	Species	ANI/dDDH/AAI (%)					
					*H. heilmannii* ASB1^T^	*H. ailurogastricus* ASB7^T^	*H. bizzozeronii* CIP105233^T^	*H. gastrocanis* sp. nov NHP19-003^T^	*H. gastrofelis* sp. nov. NHP19-012^T^	*H. felistomachi* sp. nov. NHP21-005^T^
NHP19-003^T^	2019	dog	protein-losing enteropathy	*Helicobacter* sp.	85.1/54.1/88.4	85.1/63.2/87.0	83.5/16.9/73.4	**100.0/100.0/100.0**	85.7/61.3/88.4	86.5/65.5/88.7
NHP19-012^T^	2019	cat	large-cell lymphoma	*Helicobacter* sp.	86.7/48.4/87.7	84.9/59.9/87.3	83.8/18.5/72.9	85.7/61.3/88.4	**100.0/100.0/100.0**	86.5/57.4/89.8
NHP21-005^T^	2021	cat	eosinophilic enteropathy	*Helicobacter* sp.	86.6/48.4/88.9	85.9/57.6/87.7	83.7/17.2/72.8	86.5/65.5/88.7	86.5/57.4/89.8	**100.0/100.0/100.0**
NHP22-001	2022	cat	duodenal ulcer	*Helicobacter* sp.	86.8/49.3/88.4	85.0/60.9/86.9	83.8/18.5/73.1	85.7/61.2/88.4	**98.5/98.6/98.8**	86.4/56.9/88.6
NHP19-002	2019	cat	gastric ulcer	*H. ailurogastricus*	84.6/42.1/85.8	**98.4/87.1/98.2**	83.9/18.5/73.5	85.2/56.5/87.1	85.1/52.8/87.2	86.1/53.4/87.5
NHP19-009	2019	cat	triaditis	*H. ailurogastricus*	84.7/45.4/85.6	**98.3/92.5/98.5**	83.9/17.0/73.4	85.1/57.9/87.0	85.1/57.5/87.2	85.9/53.9/87.6
NHP20-010	2020	dog	gastric cancer	*H. bizzozeronii*	83.9/15.9/72.3	84.5/18.0/73.1	**95.5/72.8/96.0**	83.6/17.5/73.5	84.7/17.2/72.8	83.6/16.4/72.8
NHP20-013	2020	dog	protein-losing enteropathy	*H. bizzozeronii*	83.3/15.3/72.6	84.3/16.8/73.8	**95.6/71.9/96.3**	83.8/16.5/73.9	84.3/17.4/73.6	83.5/16.4/73.4
NHP21-011	2021	cat	gastritis	*H. heilmannii*	**97.2/96.8/97.3**	84.6/56.6/86.4	83.4/15.8/72.8	85.1/59.2/89.1	86.8/54.9/88.1	86.5/51.6/89.3

ANI, average nucleotide identity; dDDH, digital DNA–DNA hybridization; AAI, average amino acid identity. Values in bold indicate that they are above the threshold for species identification.

### Isolation source and electron microscopic images of novel *Helicobacter* species

**Figure 2:**
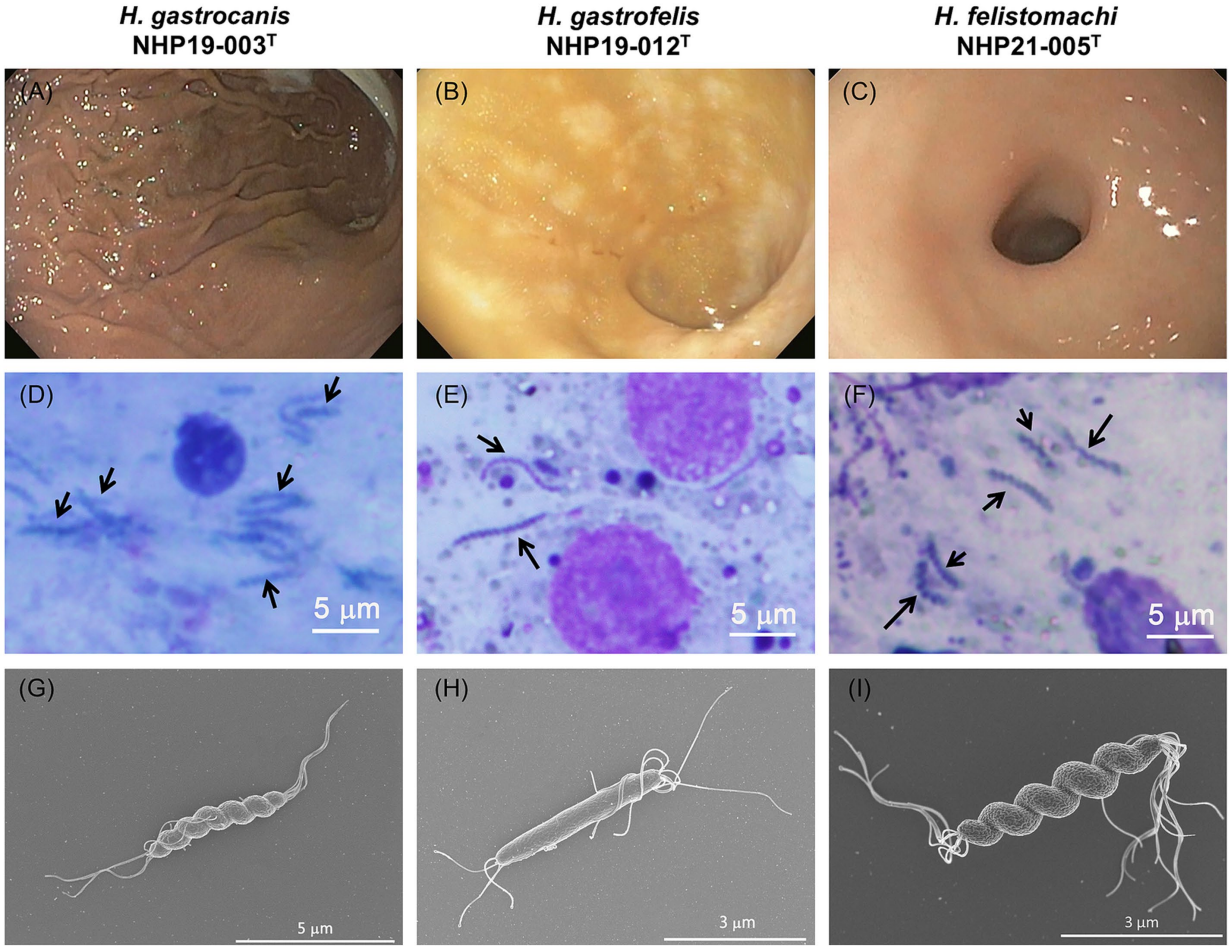
Endoscopic and histological images from NHP19-003^T^–infected dog, NHP19-012^T^–infected cat, and NHP21-005^T^–infected cat. Representative endoscopic images NHP19-003^T^–infected dog suffering from protein-losing enteropathy **(A)**, NHP19-012^T^–infected cat suffering from large-cell lymphoma **(B)**, and NHP21-005^T^–infected cat suffering from gastric lymphoma **(C)**. Giemsa staining of gastric mucosa of infected dog **(D)** and infected cat **(E,F)**. Giemsa staining showed the presence of corkscrew-like morphology of the bacteria (arrows). Scanning electron micrograph of strains NHP19-003^T^ **(G)**, NHP19-012^T^ **(H)**, and NHP21-005^T^ **(I)**. Electron microscopy showed the spiral bacilli with a characteristic twisted structure with multiple flagella at both ends.

Three strains of novel *Helicobacter* species, NHP19-003^T^, NHP19–012^T^, and NHP21-005^T^, were isolated from a dog (a 6-year-old female, suffering from protein-losing enteropathy), a cat (a 14-year-old female, suffering from large-cell lymphoma), and another cat (a nine-year-old female, suffering from eosinophilic enteropathy), respectively. No specific endoscopic findings due to *Helicobacter* species infection were observed in all three cases (Figures 2A–C). The presence of spiral-shaped bacteria was confirmed by Giemsa staining of gastric biopsies, and all strains exhibited a corkscrew-like form *in vitro* (Figures 2D–F). Electron microscopy showed that NHP19-003^T^ and NHP21-005^T^ displayed spiral bacilli with a characteristic twisted structure with multiple flagella at both ends (Figures 2G,I), while NHP19-012^T^ appeared rod-shaped without torsion after passaging (Figure 2H).

### Genomic characterization of novel *Helicobacter* species

Table 2 displays the complete genomic information of NHP19-003^T^, NHP19-012^T^, NHP21-005^T^, and three relative gastric *Helicobacters*. The complete genome of NHP19-003^T^ consists of one chromosome (Accession No. AP024814) and four plasmids (Accession No. AP024815, AP024816, AP024817 and AP024818), with a G + C content of 48.3%. The G + C content of NHP19-003^T^ was found to be slightly higher than that of *H. heilmannii* (47.8%). The genome of NHP19–012^T^ consists of one chromosome (Accession No. AP024819) and seven plasmids (Accession No. AP024820, AP024821, AP024822, AP024823, AP024824, AP024825 and AP024826, respectively), with a G + C content of 46.9%. The G + C content of NHP19-012^T^ was found to be slightly lower than those of *H. heilmannii*, *H. ailurogastricus* (47.6%) and NHP19-003^T^. The complete genome of NHP21-005^T^, consists of one chromosome (1,737,144 bp, Accession No. AP028022) and eight plasmids (Accession No. AP028023, AP028024, AP028025, AP028026, AP028027, AP028028, AP028029, and AP028030), with a G + C content of 47.1%. The G + C content of NHP21-005^T^ was found to be slightly lower than those of *H. heilmannii*, *H. ailurogastricus* and NHP19-003^T^.

**Table 2:** Complete genome information of *Helicobacter gastrocanis* sp. nov. NHP19-003^T^, *Helicobacter gastrofelis* sp. nov. NHP19-012^T^, *Helicobacter felistomachi* sp. nov. NHP21-005^T^, and three related gastric *Helicobacter* species.

Species	*H. gastrocanis* sp. nov. NHP19-003^T^	*H. gastrofelis* sp. nov. NHP19-012^T^	*H. felistomachi* sp. nov. NHP21-005^T^	*H. heilmannii* ASB1^T^	*H. ailurogastricus* ASB7^T^	*H. suis* HS1^T^
Origin	Dog	Cat	Cat	Cat	Cat	Pig
Accession no.	AP024814	AP024819	AP028022	AP026684	AP026687	AP026769
Total Length (bp)	1,539,826	1,510,356	1,737,144	1,621,963	1,580,959	1,689,191
GC content (%)	48.30	46.90	47.10	47.80	47.60	40.20
No. of CDSs	1,589	1,616	1,892	1,795	1,674	1,847
No. of rRNA	6	6	6	6	5	5
No. of tRNA	37	37	37	36	37	35
Coding ratio (%)	92.50	89.40	89.50	89.00	92.40	90.20
Plasmid (bp)	pNPH19003_1 (29,454)	pNHP19012_1 (47,061)	pNPH21005_1 (86,195)	pASB1_1 (21,547)	pASB7_1 (12,904)	pHS_1 (43,388)
	pNPH19003_2 (16,561)	pNHP19012_2 (19,511)	pNPH21005_2 (24,904)	pASB1_2 (17,441)	pASB7_2 (12,904)	pHS_2 (43,198)
	pNPH19003_3 (15,031)	pNHP19012_3 (11,414)	pNPH21005_3 (13,261)		pASB7_3 (3,243)	
	pNPH19003_4 (9,934)	pNHP19012_4 (9,367)	pNPH21005_4 (9,458)			
		pNHP19012_5 (7,455)	pNPH21005_5 (7,167)			
		pNHP19012_6 (5,732)	pNPH21005_6 (6,688)			
		pNHP19012_7 (1,769)	pNPH21005_7 (4,811)			
			pNPH21005_8 (1,835)			

**Figure 3:**
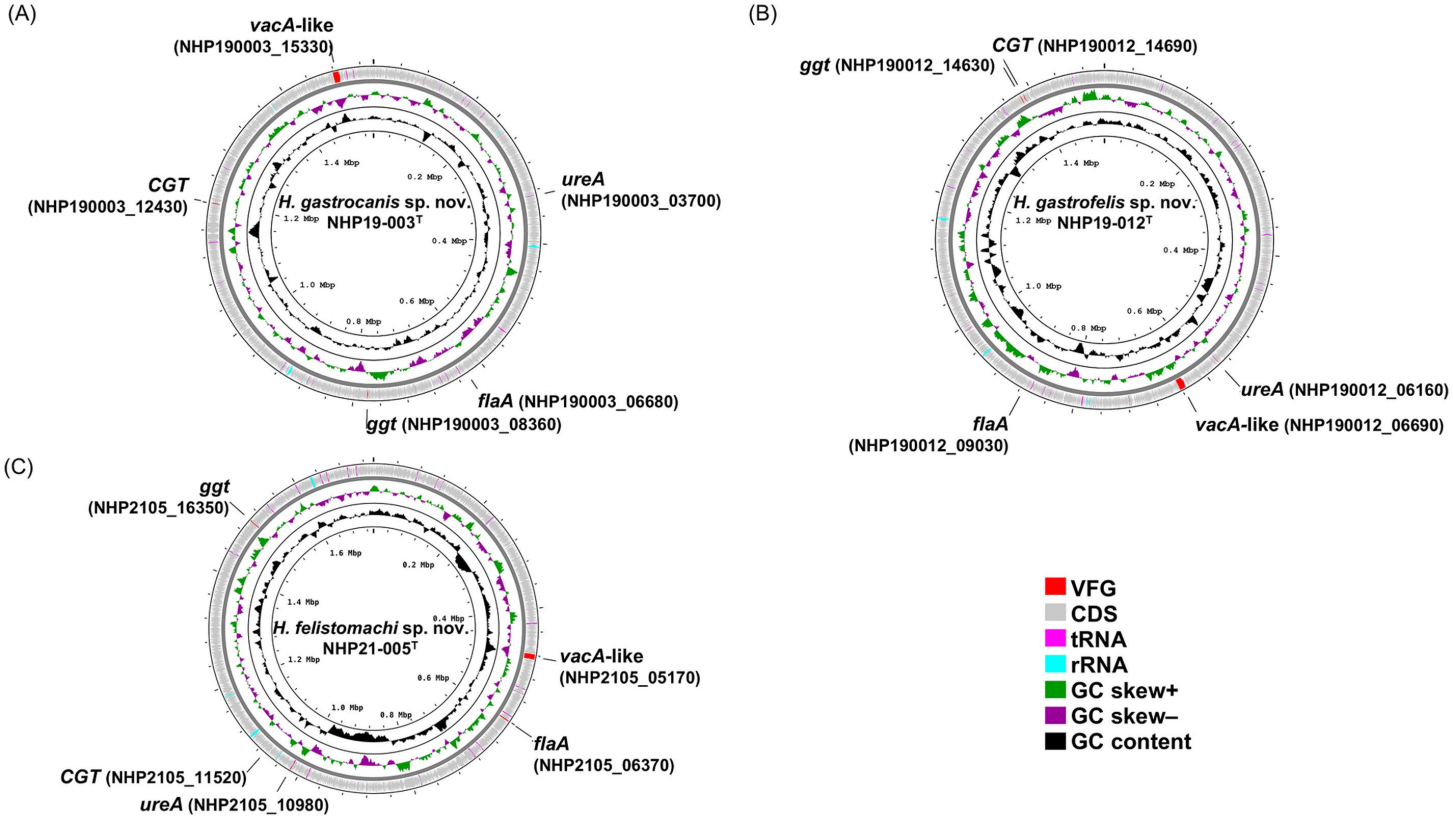
A circular genomic map of NHP19-003^T^, NHP19-012^T^, and NHP21-005^T^, comparing a circular graphical display of the distribution of genome information. The gene maps of **(A)** *Helicobacter gastrocanis* sp. nov. NHP19-003^T^; **(B)** *Helicobacter gastrofelis* sp. nov. NHP19-012^T^; and **(C)** *Helicobacter felistomachi* sp. nov. NHP21-005^T^. VFG, virulence factor gene; CDS, coding sequence.

**Figure 4:**
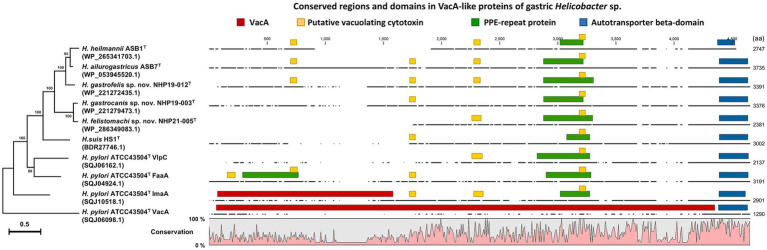
Phylogenetic tree, conserved regions, and domains in VacA of *H. pylori* ATCC43504^T^ and VacA-like proteins of *H. heilmannii* ASB1^T^, *H. ailurogastricus* ASB7^T^, *H. suis* HS1^T^, NHP19-003^T^, NHP19-012^T^, NHP21-005^T^, and *H. pylori* ATCC43504^T^. The amino acid sequences of VacA and VacA-like proteins were aligned by MAFFT version 7.49, and phylogenetic tree was constructed using RAxML-NG version 1.1.0 with an LG + G4 model and 1,000 bootstrap replicates. Numbers indicate bootstrap percentages, and the scale bar indicates the number of base substitutions per site. Conserved domains were identified using the NCBI platform’s CD-search tool. The conserved regions and identified domains were visualized using CLC Genomics Workbench Version 22.0.2.

The chromosomes of NHP19-003^T^, NHP19-012^T^ and NHP21-005^T^ as circular genome maps (Figures 3A–C). NHP19-003^T^, NHP19-012^T^ and NHP21-005^T^ all exhibited urease activity in biochemical tests using API Campy, as shown later, and genomic analysis confirmed the presence of the urease gene complex containing *ureA* genes. In addition, genes related to motility (flagellin, FlaA), colonization in host cells (cholesterol-*α*-glucosyltransferase, CGT), and induction of apoptosis in host cells (gamma-glutamyltranspeptidase, GGT), which contribute to the pathogenesis in other gastric *Helicobacter* species (Leying et al., 1992; Shibayama et al., 2003; Hsu et al., 2021), are also present in the chromosomes of NHP19-003^T^, NHP19-012^T^ and NHP21-005^T^. Vacuolating toxin A (VacA) and Cytotoxin-associated-gene A (CagA), which are virulence factors of *H. pylori*, were not found in NHP19-003^T^, NHP19-012^T^, and NHP21-005^T^, as in other gastric *Helicobacter* species. Meanwhile, the gene encoding a VacA-like protein was located in the genome of NHP19-003^T^, NHP19-012^T^ and NHP21-005^T^. *H. pylori* has three VacA-like proteins other than VacA, which are ImaA, VlpA, and FaaA and *H. suis* also has a VacA-like protein HsvA. Phylogenetic comparison of the amino acid sequences of these VacA-like proteins in gastric *Helicobacter* spp. (Figure 4) revealed that the VacA-like proteins in NHP19-003^T^, NHP19-012^T^ and NHP21-005^T^ most closely resemble those in *H. heilmannii* ASB1^T^ and *H. ailurogastircus* ASB7^T^. All VacA-like proteins lack the VacA domain shown in red in Figure 4, and the lengths of the VacA-like proteins differ from that of VacA, suggesting VacA-like proteins have functions different from those of VacA.

### Phylogenetic characterization

**Figure 5:**
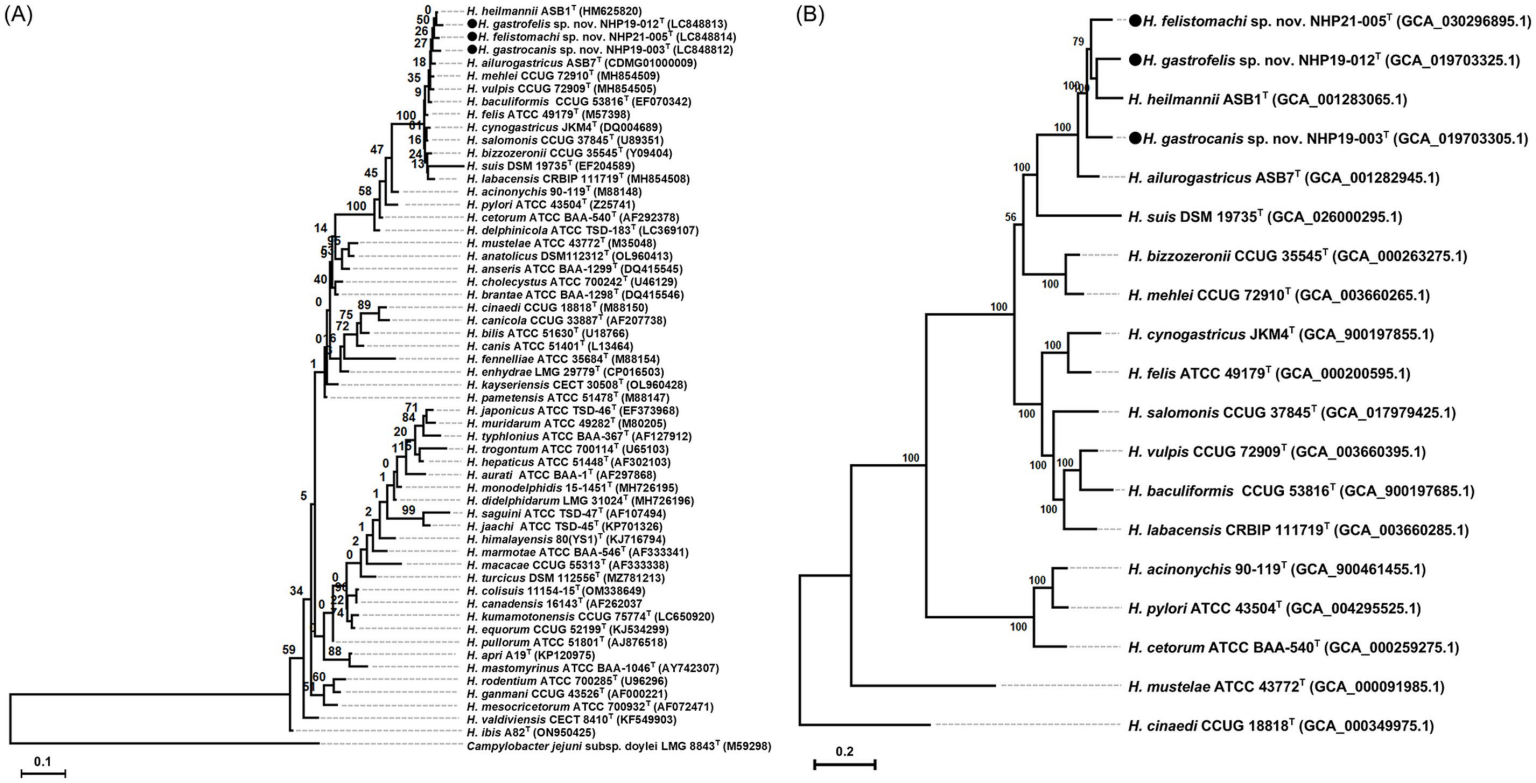
Phylogenetic trees showing the relationship of NHP19-003^T^, NHP19-012^T^ and NHP21-005^T^ to their closely related species. **(A)** Is created from 16S rRNA gene sequences of *Helicobacter* species including NHP19-003^T^, NHP19-012^T^, NHP21-005^T^ and 54 *Helicobacter* species. *Campylobacter jejuni* LMG 8841^T^ was added as an out group. The sequences were aligned by MAFFT version 7.49 and the phylogenetic tree was constructed using RAxML-NG version 1.1.0 with a GTR + G + I model and 1,000 bootstrap replicates. Numbers indicate bootstrap percentages, and the scale bar indicates the number of base substitutions per site. Circles indicated *Helicobacter* sp. strain NHP19-003^T^, NHP19-012^T^, and NHP21-005^T^, respectively. **(B)** Is generated from 230 core genes of gastric *Helicobacter* species. Core genes alignments were obtained from Roary and aligned sequences were used for the phylogenetic tree reconstruction using RAxML-NG version 1.1.0 with a GTR + G + I model and 1,000 bootstrap replicates. *H. cinaedi* was included as an out group. Numbers and the scale bar indicate bootstrap percentages and substitutions per nucleotide position, respectively. Circles indicate *Helicobacter* sp. strain NHP19-003^T^, NHP19-012^T^, and NHP21-005^T^, respectively.

The phylogenetic tree was generated from the 16S rRNA sequences of NHP19-003^T^, NHP19-012^T^, NHP21-005^T^ and 54 other *Helicobacter* sp. (Figure 5A). NHP19-003^T^, NHP19-012^T^ and NHP21-005^T^ were in the clade containing *H. bizzozeronii*, *H. cynogastricus*, *H. felis*, *H. ailurogastricus*, *H. heilmannii*, and *H. salomonis*, which are all gastric *Helicobacters* from cats and dogs. Among them, NHP19-003^T,^ NHP19-012^T^, and NHP21-005^T^ were most closely related to *H. heilmannii* ASB1^T^. The identity of the 16S rRNA sequences of NHP19-003^T^, NHP19-012^T^ and NHP21-005^T^ with *H. heilmannii* ASB1 from cats and dogs in the same clade were 98.8, 99.2 and 99.1%, respectively. This result suggested that phylogenetic analysis using 16S rRNA sequence failed to determine the taxonomic position of NHP19-003^T^, NHP19-012^T^, and NHP21-005^T^ within the genus *Helicobacter*. By phylogenetic analysis using whole genomic sequences, we successfully identified 230 genes as the core genome among gastric *Helicobacter* species and *Helicobacter cinaedi*, defined as those genes present in over 99% of the species, with an identity higher than 60%. Phylogenetic analysis revealed that NHP19-003^T^, NHP19-012^T^, and NHP21-005^T^ belonged to a clade containing *H. heilmannii* and *H. ailurogastricus*, both isolated from cat, and were clearly distinct from both species (Figure 5B).

### Phenotypic characterization

NHP19-003^T^, NHP19-012^T^ and NHP21-005^T^ grew on NHPH agar plates (Rimbara et al., 2021). Tolerance to 1% bile, 1% glycine, and 1.5% NaCl was not observed in either NHP19-003^T^, NHP19-012^T^, and NHP21-005^T^. On the other hand, catalase- and oxidase-activities were positive. Growth of NHP19-003^T^, NHP19-012^T^ and NHP21-005^T^ occurred at 37°C, but not at 25 and 42°C under microaerobic conditions (Table 3). They also grew well in microaerophilic conditions and very weakly in anaerobic conditions, but did not grow under aerobic conditions.

**Table 3:** Characteristics of *Helicobacter gastrocanis* sp. nov. NHP19-003^T^, *Helicobacter gastrofelis* sp. nov. NHP19-012^T^, *Helicobacter felistomachi* sp. nov. NHP21-005^T^, and related gastric *Helicobacter* species.

Characteristic	1	2	3	4	5	6	7	8	9	10	11	12
Cell size (uM)												
Length	4.0–7.5	3.4–6.3	2.5–7.4	3.0–6.5	3–5.5	2.3–6.7	10	10–18	5–10	5.0–7.5	5.0–7.0	2.5–5.0
Width	0.5–0.7	0.4–0.5	0.6–1.0	0.6–0.7	0.5–0.7	0.9–1.2	1	0.8–1.0	0.3	0.4	0.8–1.2	0.5–1.0
Nitrate reduction	+	+	−	+	+	−	+	+	+	+	+	−
Hydrolysis of:												
Alkaline phosphate	+	+	+	−	+	+	+	+	+	+	+	+
Indoxyl acetate	−	−	−	−	−	−	−	−	+	−	+	−
Growth at 42°C	−	−	−	−	−	−	−	−	+	−	−	−
Periplasmic fibrils	−	−	−	−	−	−	+	+	−	+	−	−
Flagella												
No. per cell	6–14	6–8	2–17	4–10	6–8	4–10	11	6–12	10–20	14–20	10–23	4–8
Distribution*	BP	BP	BP	BP	BP	BP	BP	BP	BP	BP	BP	MP

Taxa: 1, *H. gastrocanis* sp. nov. (data from this study); 2, *H. gastrofelis* sp. nov. (data from this study); 3, *H. felistomachi* sp. nov. (data from this study); 4, *H. heilmannii*; 5, *H. ailurogastricus*; 6, *H. suis*; 7, *H. baculiformis*; 8, *H. cynogasticus*; 9, *H. bizzozeronii*; 10, *H. felis*; 11, *H. salomonis*; 12, *H. pylori*. Data of taxa 4–12 was obtained from (7) and (2). *BP, Bipolar; MP, monopolar.

### MALDI-TOF MS assays

**Figure 6:**
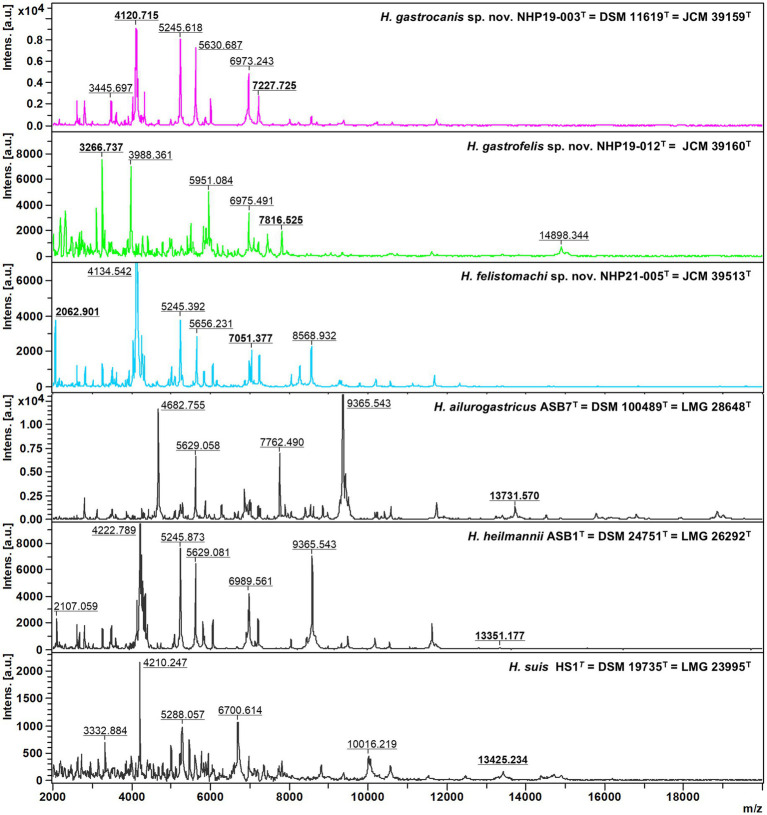
Comparative MALDI-TOF spectra profiles of type strains of novel species and related *Helicobacter* species strains obtained with flexAnalysis software. The baseline was subtracted. The y-axis shows the relative intensities of the ions, and the x-axis shows ion masses (Da). The peak at m/z approximately 13,000 pertaining to three other gastric *Helicobacter* species, *H. ailurogastricus*, *H. heilmannii*, and *H. suis* was not detected in NHP19-003^T^, NHP19-012^T^, and NHP21-005^T^.

Protein profiles of NHP19-003^T^, NHP19-012^T^ and NHP21-005^T^ generated from MALDI Biotyper (BrukerBiotyper, BrukerDaltonics) were compared with those of *H. ailurogastricus* ASB7^T^, *H. suis* HS1^T^ and *H. heilmannii* ASB1^T^ (Figure 6). The profiles of NHP19-003^T^, NHP19-012^T^ and NHP21-005^T^ differed from those of known type strains close to them. Compared with the type strain *H. ailurogastricus*, NHP19-003^T^ had two specific peaks at 4,120 and 7,227 m/z. Compared with the type strain *H. heilmannii*, NHP19-012^T^ had unique peaks at 3,266 and 7,816 m/z. Compared with the type strain *H. heilmannii*, NHP21-005^T^ had unique peaks at 2,062 and 7,051 m/z. In addition, the peak at m/z approximately 13,000 pertaining to three other gastric *Helicobacters*, was not detected in NHP19-003^T^, NHP19-012^T^ and NHP21-005^T^ making them different from other related strains.

### Prevalence of gastric *Helicobacter* species in cats and dogs in Japan

**Figure 7:**
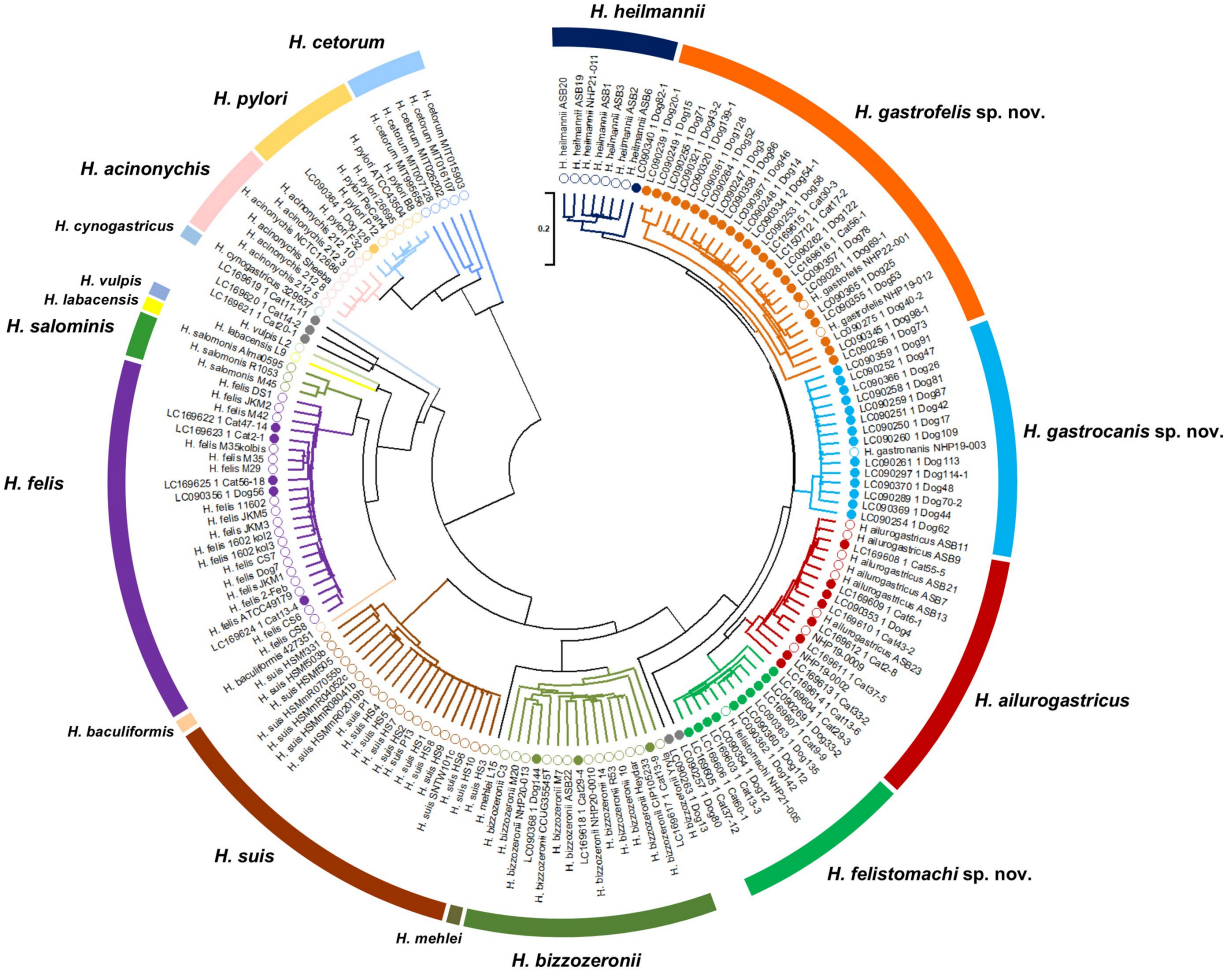
Phylogenetic tree generated from *ureAB* gene sequences of gastric *Helicobacter* species. The *ureAB* sequences shown as the GenBank no. (LCXXXXXX) from cats (*n* = 24) and dogs (*n* = 47) in Japan analyzed in previous studies (13, 14). The *ureAB* sequences obtained from nine strains analyzed in this study (NHP19-003^T^, NHP19-012^T^, NHP21-005^T^, NHP22-001, NHP19-002, NHP19-009, NHP20-010, NHP20-013, and NHP21-011), and 88 reference strains of gastric *Helicobacter* species strains whose genomic sequences were available from NCBI were added to the analysis. The accession numbers of the *ureAB* sequences used in the analysis are summarized in Supplementary Table S4. The sequences were aligned by MAFFT version 7.49 and the tree was constructed using RAxML-NG version 1.1.0 with a GTR + G + I model and 1,000 bootstrap replicates. Numbers indicate bootstrap percentages, and the scale bar indicates the number of base substitutions per site. Open circles are 88 reference strains and nine strains analyzed in this study, and closed circles are 71 samples obtained from cats and dogs in Japan in previous studies (13, 14). Light blue, orange and green open circles indicate *Helicobacter* sp. strain NHP19-003^T^, NHP19-012^T^, NHP22-001 and NHP21-005^T^, respectively. The *ureAB* sequences in light blue, orange, and green subtrees were predicted to belong to the same species as *Helicobacter* sp. strain NHP19-003^T^, NHP19-012^T^, and NHP21-005^T^, respectively.

The *ureAB* genes sequences obtained from gastric specimens of *Helicobacter*-species-infected dogs (*n* = 47) and cats (*n* = 24) in Japan analyzed in a previous study (Kubota-Aizawa et al., 2017a; Kubota-Aizawa et al., 2017b) were re-evaluated by phylogenetic analysis using the nine strains obtained in this study (Figure 7). The analysis included 90 sequences of gastric *Helicobacter* species for which genomic sequences were available from NCBI. The clades containing NHP19-003^T^, NHP19-012^T^, and NHP21-005^T^ included 14 (19.7%), 24 (33.8%) and 10 (14.1%) of the 71 sequences from dogs and cats in Japan (Table 4). The clades of known *Helicobacter* sp., including *H. heilmannii*, *H. ailurogastricus*, *H. bizzozeronii*, *H. felis,* and *H. pylori* included one (1.4%), eight (11.3%), three (4.2%), five (7.0%), and one (1.4%) sequences from dogs and cats in Japan, respectively (Table 4).

**Table 4:** Prevalence of gastric *Helicobacter* species among dogs and cats in Japan.

*Helicobacter* species	No. (%) of strains					
	Dog (*n* = 47)		Cat (*n* = 24)		Total (*n* = 71)	
*H. gastrocanis* sp. nov.	14	(29.8)	0	(0)	14	(19.7)
*H. gastrofelis* sp. nov.	21	(44.7)	3	(12.5)	24	(33.8)
*H. felistomachi* sp. nov.	5	(10.6)	5	(20.8)	10	(14.1)
*H. heilmannii*	1	(2.1)	0	(0)	1	(1.4)
*H. ailurogastricus*	1	(2.1)	7	(29.2)	8	(11.3)
*H. bizzozeronii*	1	(2.1)	2	(8.3)	3	(4.2)
*H. felis*	1	(2.1)	4	(16.7)	5	(7.0)
*H. pylori*	1	(2.1)	0	(0)	1	(1.4)
Others	2	(4.3)	3	(12.5)	5	(7.0)

The prevalence was estimated from the sequences obtained from gastric specimens of dogs (Kubota-Aizawa et al., 2017a) and cats (Kubota-Aizawa et al., 2017b) in Japan.

### Mice infection

**Figure 8:**
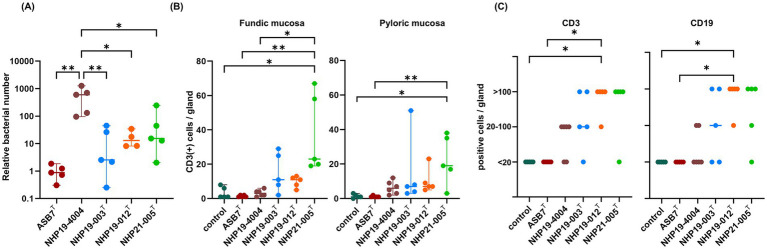
Effect of novel *Helicobacter* species infection in mouse stomachs. **(A)** Relative bacterial number of *Helicobacter* species in the mouse stomach. Bars indicate medians with 95% confidence interval. **(B)** Number of CD3(+) cells per glands in fundic and pyloric mucosa. **(C)** Number of CD3(+) cells and CD19(+) cells in the forestomach-glandular border. *P < 0.05, **P < 0.01.

**Figure 9:**
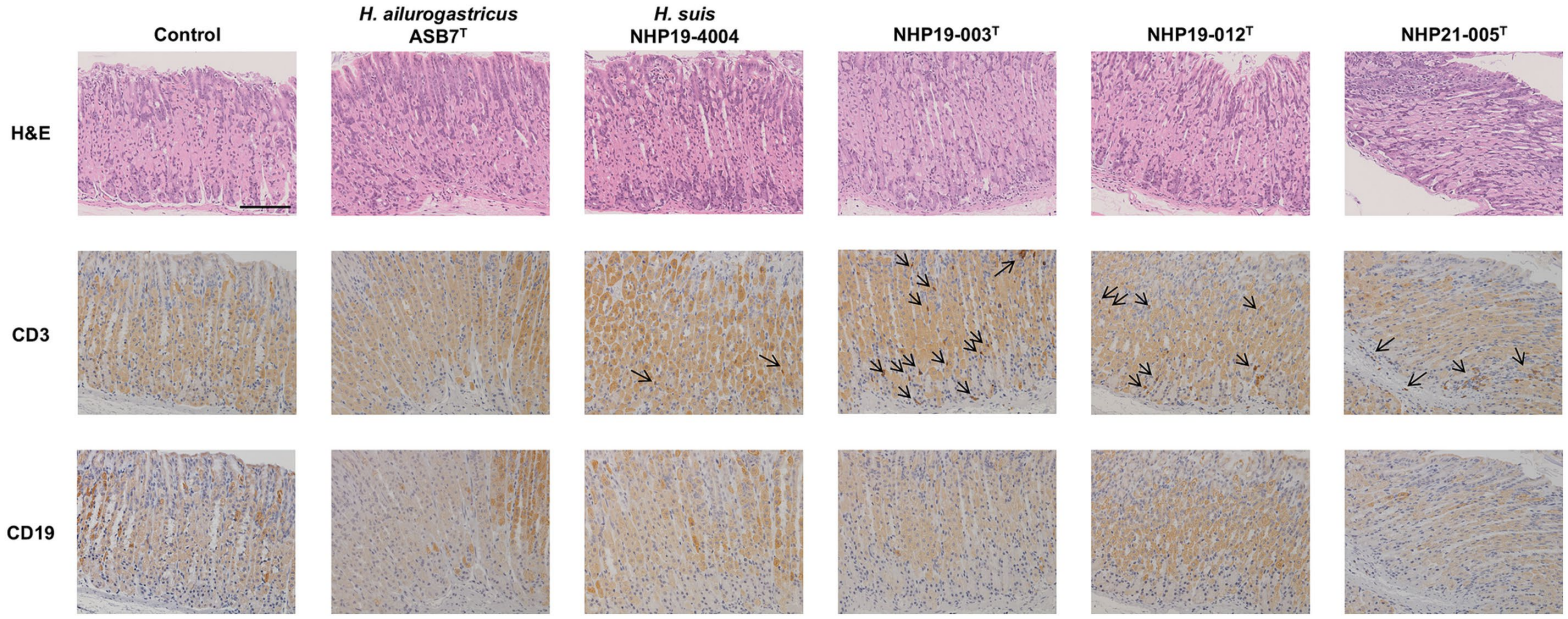
Effect of novel *Helicobacter* species infection in fundic mucosa of mouse stomachs. H&E, CD3, and CD19 staining in fundic mucosa of stomach sections of *Helicobacter* species-infected and control mice. CD3(+) lymphocytes (arrows) were observed in NHP19-003^T^, NHP19-012^T^, and NHP21-005^T^–infected mice, while most lymphocytes were mostly negative for CD19. Bar indicates 100 μm.

**Figure 10:**
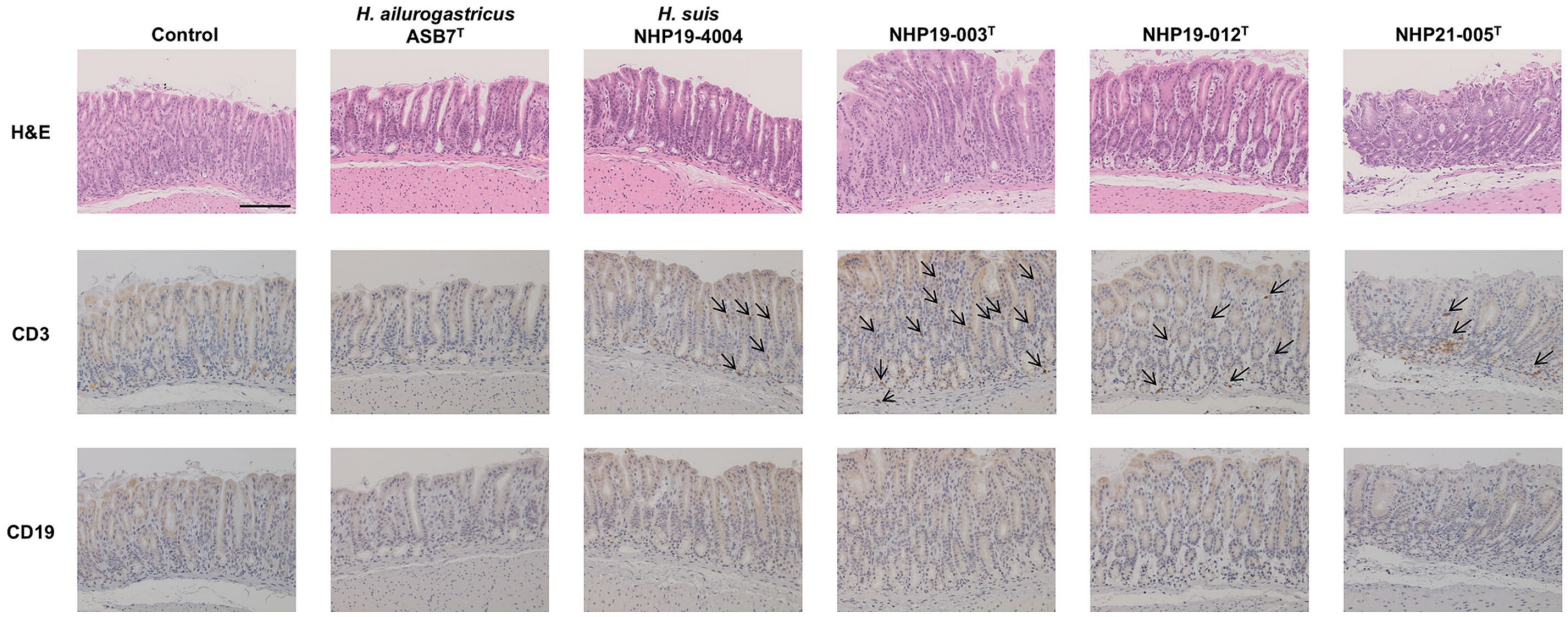
Effect of novel *Helicobacter* species infection in pyloric mucosa of mouse stomachs. H&E, CD3, and CD19 staining in pyloric (E) mucosa of stomach sections of *Helicobacter* species-infected and control mice. CD3(+) lymphocytes (arrows) were observed in NHP19-003^T^, NHP19-012^T^, and NHP21-005^T^–infected mice, while most lymphocytes were mostly negative for CD19. Bar indicates 100 μm.

**Figure 11:**
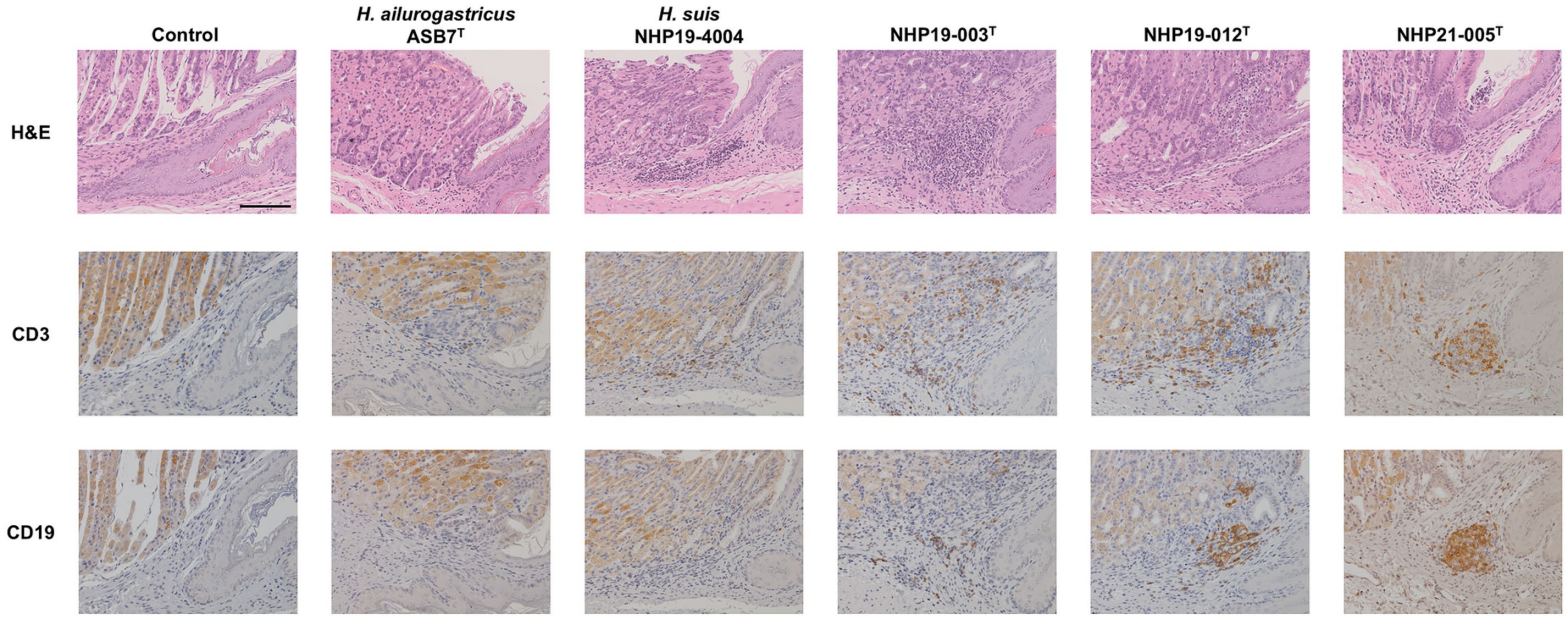
Effect of novel *Helicobacter* species infection in forestomach-glandular border of 689 mouse stomachs. H&E, CD3, and CD19 staining in the forestomach-glandular border of stomach sections from *Helicobacter* species-infected and control mice. Bar indicates 100 mm.

Mice infection was performed using *H. ailurogastricus* ASB7^T^, *H. suis* NHP19-4004, NHP19-003^T^, NHP19-012^T^ and NHP21-005^T^. Bacterial counts infecting the stomach were compared between groups and found that bacterial counts of *H. suis* NHP19-4004-infected mice were significantly higher compared to ASB7^T^, NHP19-003^T^, NHP19-012^T^ and NHP21-005^T^-infected groups (Figure 8A). Counts of CD3(+)-T cells were significantly higher in NHP21-005^T^-infected stomach compared to control, *H. ailurogastricus* ASB7^T^, and *H. suis* NHP19-4004 in fundic mucosa (Figures 8B, 9), and compared to control and *H. ailurogastricus* ASB7^T^ in pyloric mucosa (Figures 8B, 10). Counts of CD3(+)-T cells of NHP19-003^T^ and NHP19-012^T^ tended to be higher than control, *H. ailurogastricus* ASB7^T^, and *H. suis* NHP19-4004-infected mucosa without statistical significance (Figure 8B). Almost no CD19(+)-B cells were found in control and infected gastric mucosa (Figures 8D, 10) except the forestomach-glandular border (Figure 11). In the forestomach-glandular border, lymphocyte aggregation was found in *H. suis* NHP19-4004, NHP19-003^T^, NHP19-012^T^ and NHP21-005^T^-infected mice and number of CD3(+)-T cells and CD19(+)-B cells were significantly higher in NHP19-012^T^-infected mice compared to control and *H. ailurogastricus* ASB7^T^-infected mice (Figure 8C).

## Discussion

### Genomic and pathogenetic characteristics of three novel *Helicobacter* species

*Helicobacter* species are important causative agents of zoonotic diseases. In previous studies, it was suspected that dogs and cats might be infected with novel *Helicobacter* species. In this study, nine strains of *Helicobacter* spp. were isolated from gastric biopsy tissues of dogs and cats and identified through whole genome analysis. The nine strains include three known *Helicobacter* spp. *H. ailurogastricus*, *H. heilmannii*, and *H. bizzozeronii*. In addition, three strains NHP19-003^T^, NHP19-012^T^, and NHP21-005^T^ were isolated in pure culture from the gastric biopsy of a dog and a cat, respectively, as novel *Helicobacter* species. Whole genome sequencing indicated that the ANI value was less than 95% identity between the genome of the three novel *Helicobacter* species and those of all other known species in the *Helicobacter* genus. The identity of 16S rRNA sequences between the genome of the three novel *Helicobacter* species and those of all other known species was more than 98.5%, consistent with previous reports showing that *Helicobacter* species cannot always be identified to species by 16S rRNA analysis alone (Dewhirst et al., 2005). Additionally, MALDI-TOF MS assay results showed that the peak at m/z approximately 13,000 pertaining to other gastric *Helicobacter* species was not detected in NHP19-003^T^, NHP19-012^T^ and NHP21-005^T^. Based on these results, NHP19-003^T^, NHP19-012^T^ and NHP21-005^T^ are proposed as novel *Helicobacter* sp., named *Helicobacter gastrocanis* (NHP19-003^T^), *Helicobacter gastrofelis* (NHP19-012^T^) and *Helicobacter felistomachi* (NHP21-005^T^), respectively.

Whole genome sequencing revealed that *H. gastrocanis* sp. nov., *H. gastrofelis* sp. nov. and *H. felistomachi* sp. nov. strains had CGT, GGT, and VacA-like proteins, which have been reported as virulence factors in other *Helicobacter* spp. Among them, VacA-like protein, an autotransporter protein that transports the passenger domain of itself using the type V secretion system, is common to gastric *Helicobacter* species. *H. pylori* possess three autotransporter proteins other than VacA, ImaA, VlpC, and FaaA, which have been shown to contribute to colonization (Sause et al., 2012; Radin et al., 2013). *H. heilmannii* and *H. ailurogastricus* also have VacA-like proteins, the contribution of which to pathogenicity has been discussed (Baele et al., 2008). The VacA-like proteins of *H. gastrocanis* sp. nov., *H. gastrofelis* sp. nov. and *H. felistomachi* sp. nov. most resemble those of *H. heilmannii* and *H. ailurogastricus* and possess conserved domains among VacA-like proteins of ImaA, VlpC, and FaaA suggesting their contribution to colonization, although further analysis is needed to elucidate the functions of VacA-like proteins.

Three novel *Helicobacter* spp. were infected in mice. The results showed that inflammatory response in the stomach was induced after 1 month of infection. *H. heilmannii* is reported to cause mucous metaplasia in mice 9 weeks post-infection (Liu et al., 2014), while it is suggested in this study that *H. heilmannii* is not prevalent *Helicobacter* species in dogs and cats in Japan. On the other hand, *H. ailurogastricus* is prevalent in Japan; however, the pathogenicity of *H. ailurogastricus* has been reported to be less severe compared to *H. heilmannii* (Joosten et al., 2016). In this study, we compared the pathogenetic severity of three novel *Helicobacter* spp. with that of *H. ailurogastricus* and found that the severity of the response was generally greater compared to that induced by *H. ailurogastricus* ASB7^T^. However, since only a single strain was tested for each species, the observed differences in severity might reflect strain-specific variability rather than inherent differences between *Helicobacter* spp. In *H. pylori*, VacA is known as a virulence factor. While all strains of *H. pylori* have the *vacA* gene, different strains produce various types of VacA, including both vacuolated and non-vacuolated forms of VacA. Further studies, including testing multiple strains from each novel *Helicobacter* sp., are necessary to verify the pathogenic level among novel *Helicobacter* spp., although it is evident that they can induce gastric inflammation in mice.

### Prevalence of three novel *Helicobacter* species infecting the stomachs of dogs and cats in Japan

It has been reported that *H. heilmannii* accounts for more than 60% of the *Helicobacter* spp. detected in dogs in Europe and Korea (Wiinberg et al., 2005; Amorim et al., 2015). In Japan, testing the *ureAB* gene sequences of gastric specimens obtained from dogs and cats revealed a prevalence of unidentified *Helicobacter* spp. of 42% (21/50 samples), suggesting the presence of the novel *Helicobacter* species (Kubota-Aizawa et al., 2017a). Comparison of the *ureAB* gene sequences of the three strains of *Helicobacter gastrocanis* sp. nov., *Helicobacter gastrofelis* sp. nov. and *Helicobacter felistomachi* sp. nov. with the unidentified sequences obtained in the previous study showed that most of the unidentified sequences were classified as either *H. gastrocanis* sp. nov., *H. gastrofelis* sp. nov. or *H. felistomachi* sp. nov. Of the *Helicobacter* species detected in dogs and cats, the detection rates of the three novel strains isolated in this study accounted for 19.7, 33.8 and 14.1%, respectively. Therefore, it is suggested that *H. gastrocanis* sp. nov., *H. gastrofelis* sp. nov. and *H. felistomachi* sp. nov. found in this study are the dominant *Helicobacter* spp. infecting dogs and cats in Japan. Compared to humans and birds, pet animals such as dogs and cats rarely move between countries, and the regional characteristics of the *Helicobacter* spp. infecting their stomachs are considered to be very strong. *H. suis*, which infects pigs is known to be the most prevalent non-*H. pylori Helicobacter* spp. infecting human stomachs and causing gastric diseases such as gastric MALT lymphoma (Nakamura et al., 2020; Rimbara et al., 2021). Meanwhile gastric *Helicobacter* species infecting cats and dogs have also been known to infect human stomachs, and their association with gastric diseases has been suggested (Kusters and Kuipers, 1998; Montijo-Barrios et al., 2023; Sano et al., 2023; Taillieu et al., 2023). Furthermore, identical *H. pylori* strains have been detected in the stomachs of domesticated dogs and their owner, proving the transmission of *Helicobacter* spp. between humans and dogs (Kubota-Aizawa et al., 2021). Investigating *Helicobacter* spp. infecting dogs and cats at the genome level is expected to provide useful information to clarify the infection route of *Helicobacter* species as causative agents of zoonotic diseases.

## Conclusion

We identified three novel *Helicobacter* spp. in cats and dogs in Japan. The three novel species were shown to be pathogenetic by mice infection experiments, and the severity of the pathogenicity was higher compared to that of the known *Helicobacter* sp. *H. ailurogastricus*. We also demonstrated that the three novel species are prevalent gastric *Helicobacter* species in dogs and cats in Japan. Infection of gastric *Helicobacter* species possibly contributes to gastric diseases not only in dogs and cats but also in humans because *Helicobacter* infections have been reported in humans with gastric diseases. This study provides significant basic information to understand *Helicobacter* infection in pet animals and humans.

### Description of *Helicobacter gastrocanis* sp. nov., *Helicobacter gastrofelis* sp. nov.” and *Helicobacter felistomocahi* sp. nov.

*“Helicobacter gastrocanis”* (gas.tro.ca’nis. Gr. fem. n. *gaster*, stomach; L. masc. n. *canis*, a dog; N.L. gen. n. *gastrocanis*, of the stomach of a dog).

Cells are gram-negative, tightly coiled spirals with several turns, approximately 4.0–7.5 μm long and 0.5–0.7 μm wide. There are no periplasmic fibrils. Coccoid cells predominate in older cultures and cells are non-sporulating. Moreover, these cells are motile by means of tufts of 6 to 14 sheathed blunt-ended flagella at both ends. Growth is observed on Mueller-Hinton agar and Brucella agar supplemented with 20% fetal bovine serum, Vitox supplement (Oxoid), and 0.05% HCl. Weak growth is observed on Brucella agar supplemented with10% defibrinated horse blood. The cells grow well in microaerophilic conditions and very weak in anaerobic conditions, but not in aerobic conditions. Growth occurs at 37°C and not at 25°C or 42°C. No growth was observed on the media supplemented with 1% glycine or 1% bile. In addition, the cells were oxidase-, catalase- and urease-positive. They reduce triphenyl tetrazolium chloride and nitrate but do not hydrolyse hippurate and indoxyl acetate. They also exhibit *γ*-glutamyl transferase, pyrrolidonyl arylamidase, L-arginine arylamidase, and alkaline phosphatase activities. However, they do not exhibit L-aspartate arylamidase activities.

The type strain, NHP19-003^T^ (=JCM 39159^T^ = DSM 111619^T^), was isolated from the gastric mucosa of a dog suffering from protein-losing enteropathy in Tokyo, Japan. The G + C DNA content of the type strain is 48.3% and the complete genome analysis showed that the bacterium has a 1.5-Mb chromosome and four plasmids.

*“Helicobacter gastrofelis”* (gas.Tro.fe’ris. Gr. Fem. n. *gaster*, stomach; L. masc. n. *felis*, a cat; N.L. gen. n. *gastrofelis*, of the stomach of a cat).

Cells are gram-negative, tightly coiled spirals with several turns, approximately 3.4–6.3 μm long and 0.4–0.5 μm wide. There are no periplasmic fibrils. Coccoid cells predominate in older cultures and cells are non-sporulating. Moreover, these cells are motile by means of tufts of 6 to 8 sheathed blunt-ended flagella at both ends of the cells. Growth is observed on Mueller-Hinton agar and Brucella agar supplemented with 20% fetal bovine serum, Vitox supplement (Oxoid), and 0.05% HCl. Weak growth is observed on Brucella agar supplemented with 10% defibrinated horse blood. The cells grow well in microaerophilic conditions and very weakly in anaerobic conditions, but not in aerobic conditions. Growth occurs at 37°C and not at 25°C or 42°C. No growth was observed on the media supplemented with 1% glycine or 1% bile. In addition, the cells were oxidase-, catalase- and urease-positive. They reduce triphenyl tetrazolium chloride and nitrate but do not hydrolyse hippurate and indoxyl acetate. They also exhibit γ-glutamyl transferase, pyrrolidonyl arylamidase, L-arginine arylamidase, L-aspartate arylamidase, and alkaline phosphatase activities.

The type strain, NHP19-012^T^ (=JCM 39160^T^), was isolated from a cat suffering from large-cell lymphoma in Tokyo, Japan. The G + C DNA content of the type strain is 46.9%, and the complete genome analysis showed that the bacterium has a 1.5-Mb chromosome and seven plasmids.

*“Helicobacter felistomachi”* (fe’ris. stomachi. L. masc. n. *felis*, a cat; L. masc. n. *stomachi*, stomach; N.L. gen. n. *feistomachi*, of the stomach of a cat).

Cells are gram-negative, tightly coiled spirals with several turns, approximately 2.5–7.4 μm long and 0.6–1.0 μm wide. There are no periplasmic fibrils. Coccoid cells predominate in older cultures and cells are non-sporulating. Moreover, these cells are motile by means of tufts of 2 to 17 sheathed blunt-ended flagella at both ends. Growth is observed on Mueller-Hinton agar and Brucella agar supplemented with 20% fetal bovine serum, Vitox supplement (Oxoid), and 0.05% HCl. Weak growth is observed on Brucella agar supplemented with 10% defibrinated horse blood. The cells grow well in microaerophilic conditions and very weak in anaerobic conditions, but not in aerobic conditions. Growth occurs at 37°C and not at 25°C or 42°C. No growth was observed on the media supplemented with 1% glycine or 1% bile. In addition, the cells were oxidase-, catalase- and urease-positive. They do not reduce triphenyl tetrazolium chloride and nitrate and hydrolyse hippurate and indoxyl acetate. They also exhibit γ-glutamyl transferase, pyrrolidonyl arylamidase, L-arginine arylamidase, and alkaline phosphatase activities. However, they do not exhibit L-aspartate arylamidase activities.

The type strain, NHP21-005^T^ (=JCM 39513^T^), was isolated from the gastric mucosa of a cat suffering from eosinophilic enteropathy in Tokyo, Japan. The G + C DNA content of the type strain is 47.1% and the complete genome analysis showed that the bacterium has a 1.7-Mb chromosome and eight plasmids.

## Data Availability

The GenBank/EMBL/DDBJ accession numbers of the genome of *Helicobacter gastrocanis* sp. nov. NHP19-003^T^ (=JCM 39159^T^=DSM 111619^T^), *Helicobacter gastrofelis* sp. nov. NHP19-012^T^ (=JCM 39160^T^) and *Helicobacter felistomachi* sp. nov. NHP21-005^T^ (=JCM 39513^T^), *H. ailurogastricus* NHP19-002 and NHP19-009, *H. bizzozeronii* NHP20-010 and NHP20-013, and *H. heilmannii* NHP21-011 are AP024814, AP024819, AP028022, GCA_030270085.1, GCF_030270105.1, GCF_030270125.1, GCA_036248205.1, and GCF_030270145.1, respectively, under Bioproject accession number PRJDB8704.

## Footnotes

^1^ https://github.com/marbl/canu

^2^ http://last.cbrc.jp

^3^ https://github.com/isovic/racon

^4^ https://github.com/broadinstitute/pilon

^5^ https://dfast.nig.ac.jp

^6^ https://github.com/widdowquinn/pyani

^7^ https://github.com/endixk/ezaai

^8^ http://ggdc.dsmz.de
